# Controlling risky behavior associated with AIDS: the role of social support, family functioning, self-efficacy and AIDS risk perception

**DOI:** 10.1186/s40359-022-00839-z

**Published:** 2022-05-23

**Authors:** Ali Zakiei, Ebrahim Norouzi‬, Seyed Ramin Ghasemi, Saeid Komasi, Masoumeh Rostampour, Habibolah Khazaie

**Affiliations:** 1 Sleep Disorders Research Center, Kermanshah University of Medical Sciences, Kermanshah, Iran; 2 Social Development and Health Promotion Research Center, Kermanshah University of Medical Sciences, Kermanshah, Iran; 3 Department of Neuroscience and Psychopathology Research, Mind GPS Institute, Kermanshah, Iran; 4 Department of Psychiatry, Student Research Committee, Kurdistan University of Medical Sciences, Sanandaj, Iran

**Keywords:** Acquired immunodeficiency syndrome, Dangerous behavior, Family characteristics, Risk factors, Self-efficacy

## Abstract

**Objectives:**

We believe that major steps can be taken towards Acquired Immunodeficiency Syndrome (AIDS) prevention through identifying the relevant factors that are apt to predict risky behavior. The main purpose of the present study was to analyze and evaluate the relationship of social support, family functioning, self-efficacy and AIDS risk perception to controlling risky behavior associated with AIDS.

**Methods:**

To conduct this cross-sectional study, 765 subjects (59% female) were selected from the youth inhabiting the western provinces of Iran through cluster sampling. Five questionnaires were used: AIDS risk perception, self-efficacy in controlling risky behavior associated with AIDS, controlling risky behavior associated with AIDS, the multidimensional scale of perceived social support, and the family assessment device.

**Results:**

The results demonstrated that all two models enjoyed acceptable fitness, and the mediating roles of self-efficacy and AIDS risk perception were confirmed. Moreover, family functioning and perceived social support together could predict 20% of the variance of controlling risky behavior associated with AIDS. The results also indicated that family functioning with a standardized coefficient of − 0.24 and self-efficacy in controlling risky behavior associated with AIDS with a standardized coefficient of 0.58 could predict the controlling risky behavior associated with AIDS (*p* < 0.01).

**Conclusions:**

Our findings suggest that self-efficacy and AIDS risk perception play major roles in controlling risky behavior associated with AIDS. Therefore, it is recommended that families and psychologists promote self-efficacy in order to prevent the occurrence of high-risk behaviors.

## Introduction

AIDS is still considered as one of the most prominent health problems worldwide in a way that the statistics announced in 2007 indicated that the various rates of AIDS infection were as follows: 3–7.2 million annually [1], given the alarming status of AIDS in Iran, the Secretary of Health declared that 46.5% of HIV-stricken people in Iran belonged to the 25–34 age group [2]. Moreover, according to the documentation provided by the Iranian National Center for AIDS Prevention, the rate of AIDS infection was 30,727 patients [3]. Researchers believe that one of the factors that influences the occurrence and rate of risky behavior is the lack of social support, although few studies have been conducted in this regard, and there is no consensus on its role [4–7], but its relationship to drug use [8] and AIDS infection [9] has been pointed to in particular, since social support causes one to cope with stressful events of life [10], though one’s perception of it depends on one’s social and personal conditions [11]. It seems that the focus of effective models on risky behavior associated with AIDS is not solely on one variable. A review of previous studies and models shows that these behaviors are influenced by factors such as (social) community, cognitive, interpersonal and personal factors [12]. Previous studies have pointed to variables such as knowledge, awareness, personality, social environment and individual perception [12–14]. The role of family, as one of the determinants of risky behavior associated with AIDS and drug use, has been mentioned in numerous studies [15–21]. Other studies have shown that low perception of risk can be accompanied by displaying risky behavior associated with AIDS, sexual risky behavior in particular, such as unprotected sex [22–24].

One can change one’s behavior. In this regard, Albert Bandura (1986) considers self-efficacy as a major factor in changing behavior [25], and according to the social cognitive theory (SCT), the self-efficacy beliefs are regarded as the most important factors in changing behavior [26]. Self-efficacy can be affected by factors such as family functioning [27]. Early experiences of self-efficacy are concentrated in the family. Actually, a wide network of family psychosocial structures may affect self-efficacy beliefs [27, 28]. Furthermore, the role of self-efficacy has been proven in relation to the AIDS preventive behavior [29–33], so that it has been referred to as the strongest contributing factor [14, 34–36]. But the key questions are, ‘how can these variables reduce risky behavior?’, and ‘what is the mechanism of the effects of family functioning and social support on risky behavior like?’.

Several theories about health-promoting behaviors have already been proposed by researchers, which were also considered in this study, including social-cognitive theory [37], protection motivation theory [38], and health belief model [39]. There is not sufficient research about controlling risky behavior associated with AIDS, and major steps need to be taken towards AIDS prevention through identifying the relevant factors that are suitable to predict risky behavior. So, the main purpose of the present study was to predict and evaluate the relationship between social support, family functioning, self-efficacy and AIDS risk perception with controlling risky behavior associated with AIDS.

## Methods

### Study design and participants

The statistical population of the present cross-sectional study consisted of the whole youth inhabiting the western provinces of Iran in 2016 (in May and June**)**. The inclusion criteria were to be in the 18–35 age range, a five-year residency in these provinces at the least, and studying for at least six whole academic years and people who did not want to collaborate in the study were excluded. Samples were selected using cluster sampling. To do so, three provinces (Kermanshah, Kurdistan, and Hamadan) were randomly chosen from five western provinces in Iran. Then, 250 subjects were selected from each of three provincial capitals according to the municipal zoning using Cochran's sample size formula. Additionally, given the likelihood of anticipated sample dropouts and lack of cooperation, 800 questionnaires were distributed among the target subjects residing in the three provincial capitals. To this end, the researchers walked around the cities, detailed explanations of how to complete the questionnaires were supplied, and the participants were requested to ask for more clarification in case of encountering problems filling out the questionnaires. Additionally, they were assured that their information would remain confidential, and their informed consent was taken. Further, the questionnaires were completed individually and collectively in the presence of the researchers. Finally, 765 questionnaires were collected and then analyzed.

This study was based on the findings of the research plan no. 94321, registered in Kermanshah University of Medical Sciences and licensed by the Research Ethics Committee to conduct the study. It should be noted that the study was approved by the Ethics Committee of Kermanshah University of Medical Sciences (KUMS.REC.1395.69).

### Study instrument and variables assessment

#### AIDS risk perception questionnaire

This 13-item tool was scored on a five-point Likert scale (0 = totally disagree, 1 = disagree, 2 = partly agree, 3 = agree, 4 = totally agree). The results of analyzing the reliability of the questionnaire demonstrated that there was a 0.70 correlation coefficient between the first and second stages of test–retest reliability, and Cronbach's alpha was 0.70. Moreover, the validity and reliability of this tool have been confirmed [12]. The respondent’s score in this questionnaire indicates the degree of AIDS risk perception. Total score ranges from 0 to 56, which higher scores show more perception of AIDS risk [40], and its Cronbach’s alpha was 0.79 in the present research.

#### Questionnaire for self-efficacy in controlling risky behavior

This 13-item tool was scored on a six-point Likert scale (0 = not at all, 1 = very little, 2 = little, 3 = to some extent, 4 = much, 5 = very much). The results of analyzing the reliability of the questionnaire indicated that there was a 0.91 correlation coefficient between the first and second stages of test–retest reliability, and Cronbach's alpha was 0.85. Moreover, the validity and reliability of this tool have been confirmed [12], and its Cronbach’s alpha was 0.80 in the present research. Questionnaire of Self-Efficacy in Controlling Risky Behavior has one factor. The obtained score is between 13 and 65, which a higher score means more Self-Efficacy in Controlling Risky Behavior [40].

#### Questionnaire for controlling risky behavior associated with AIDS

This 14-item tool was scored on a six-point Likert scale (0 = not at all, 1 = very little, 2 = little, 3 = to some extent, 4 = much, 5 = very much). The results of analyzing the reliability of the questionnaire showed that there was a 0.77 correlation coefficient between the first and second stages of test–retest reliability, and Cronbach's alpha was 0.77. Further, the validity and reliability of this tool have been confirmed [12], and its Cronbach’s alpha was 0.78 in the present research. The respondent’s score in this questionnaire ranges from 0 to 70, which a higher score indicates more control on risky behavior [40].

#### The multidimensional scale of perceived social support

In this 12-item scale, developed by Zimet et al. (1990), three dimensions of family support, support from friends, and support from others are measured [41]. Further, each dimension of this scale consists of four items, scored on a seven-point Likert scale (1 = totally disagree…, 7 = totally agree), which a higher score indicates more social support. The values of Cronbach's alpha for support from others, family support and support from friends were 0.91, 0.87, and 0.85, respectively.

#### Family assessment device (FAD)

This 60-item tool, formulated based on McMaster’s pattern, determines the structural, occupational, and interactive characteristics of families based on McMaster’s pattern. Further, six dimensions of family functioning are specified in this tool: problem solving, communication, roles, affective responses, affective involvement, and behavior control. It also includes a subscale pertaining to the general functioning of families. Scoring is based on Likert scale, and lower scores are indicative of healthier functioning. Further, a reliability of 0.72 to 0.92 has been reported in this questionnaire [42], and its validity and reliability have been confirmed in various studies [43].

### Statistical analysis

The obtained data are analyzed using SPSS-18, SPSS Inc, Chicago, Illinois, USA and AMOS-18 software application and structural equation modelling method (SEM) based on the variables of the model. Based on the obtained model, we can foresee which variables have the capability of predicting the risky behaviors. Two models were assessed in the present study: AIDS risk perception and self-efficacy in controlling risky behavior associated with AIDS were examined in the first and second model.

The current correlational study was a structural equation modeling. Descriptive and inferential statistical methods were used in the present study. These methods include mean and standard deviation method to describe the status of variables, simple correlation coefficient (Pearson) between variables to examine the relationship between variables and related test hypotheses (such as the linearity of the relationship between independent and dependent variables), other methods include statistical analysis to study structural equation modeling assumptions, structural equation modeling method to test the models in the present research, Macro bootstraping analysis of indirect relationships to investigate indirect effect of family functioning and social support in controlling risky behavior associated with AIDS. Data analysis was performed using SPSS-19 and amos-19 statistical software.

## Results

Of the 765 participants who entered the study, 59% (448 subjects) were female, and the average age of the entire sample was 27.5 ± 92.33. Socio- demographic information of participants are shown in Table 1.

**Table 1:** Socio- demographic characteristics of participants included in analyses

Characteristics	Frequency (N)	Percent (%)
Age (years)		
18–25	289	37.8
25–35	476	62.2
Sex		
Female	448	59
Male	317	41
Education		
Middle school	78	10.2
High school	274	35.8
Bachelor	320	41.8
Postgraduate	93	12.2
Occupation		
Employed	298	39
Unemployed & housewife	320	41.8
Student	136	17.8
Unresponsiveness	11	1.4
Marital status		
Never married	360	47.1
Married	398	52
Previously married	7	0.9

The mean and standard deviation of the main variables of the study as well as ages in each gender are shown in Table 2.

**Table 2:** The mean and standard deviation of the main variables of the research

	Male Mean ± SD	Female Mean ± SD	Total Mean ± SD	*p* value
Behavior controlling	58.05 ± 9.43	59.26 ± 9	56.36 ± 9.79	0.001
Self-efficacy	50.62 ± 8.67	51.22 ± 8.88	49.84 ± 8.12	0.001
Risk perception	41.89 ± 6.54	42.90 ± 5.62	40.38 ± 7.46	0.001
Family function	132.97 ± 18.24	130.07 ± 17.58	137.25 ± 18.37	0.001
Social support	30.17 ± 9.52	31.19 ± 9.35	28.66 ± 9.57	0.001
Age	27.92 ± 5.33	27.51 ± 5.27	28.50 ± 5.39	0.110

As shown in Table 2, there was a significant difference between men and women in terms of controlling risky behavior, self-efficacy in controlling risky behavior associated with AIDS, and AIDS risk perception, i.e., the mean of each of these variables was the highest among women. The results showed that there was not a significant difference between the demographic information, including age, education, occupation and socioeconomic status, and each of the variables of controlling risky behavior, self-efficacy in controlling risky behavior associated with AIDS, and AIDS risk perception.

The matrix of correlation coefficients between the variables under study is shown in Table 3. As can be seen, the highest correlations were between self-efficacy in controlling risky behavior associated with AIDS and controlling risky behavior (r = 0.71) and between affective involvement and communication (r = 0.66).

**Table 3:** The correlation coefficient matrix

	1	2	3	4	5	6	7	8	9	10	11	12	13
Behavior controlling	1												
Self-efficacy	0.71**	1											
Risk perception	0.52**	0.42**	1										
Others support	0.27**	0.26**	0.21**	1									
Family support	0.26**	0.26**	0.21**	0.62**	1								
Friends support	0.13**	0.16**	0.12**	0.40**	0.39**	1							
Problem solving	− 0.23**	− 0.18**	− 0.21**	− 0.38**	− 0.46**	− 0.21**	1						
Communication	− 0.25**	− 0.23**	− 0.30**	− 0.36**	− 0.42**	− 0.20**	0.56**	1					
Role function	− 0.27**	− 0.21**	− 0.22**	− 0.38**	− 0.46**	− 0.26**	0.52**	0.47**	1				
Emotional response	− 0.23**	− 0.21**	− 0.24**	− 0.37**	− 0.45**	− 0.18**	0.51**	0.47**	0.49**	1			
Emotional Involvement	− 0.21**	− 0.17**	− 0.22**	− 0.34**	− 0.44**	− 0.15**	0.47**	0.50**	0.66**	0.48**	1		
Behavior control	− 0.17**	− 0.18**	− 0.21**	− 0.35**	− 0.40**	− 0.22**	0.45**	0.37**	0.47**	0.37**	0.52**	1	
Overall function	− 0.24**	− 0.25**	− 0.26**	− 0.43**	− 0.50**	− 0.23**	0.69**	0.61**	0.64**	0.53**	0.60**	0.60**	1

**Correlation is significant at the 0.01 level (2-tailed)

### Model 1: investigating the mediating role of AIDS risk perception

First, a model was designed in the absence of the mediating variables. The results demonstrated that family functioning and perceived social support together could predict 20% of the variance of controlling risky behavior associated with AIDS. In addition, the results showed that family functioning with a standardized coefficient of − 0.30 (*p* < 0.001) and perceived social support with a standardized coefficient of 0.17 (*p* < 0.02) could predict the variable of controlling risky behavior associated with AIDS. To investigate the mediating role of AIDS risk perception in the relationship of family functioning and perceived social support to controlling risky behavior associated with AIDS, a model was formulated.

**Fig. 1:**
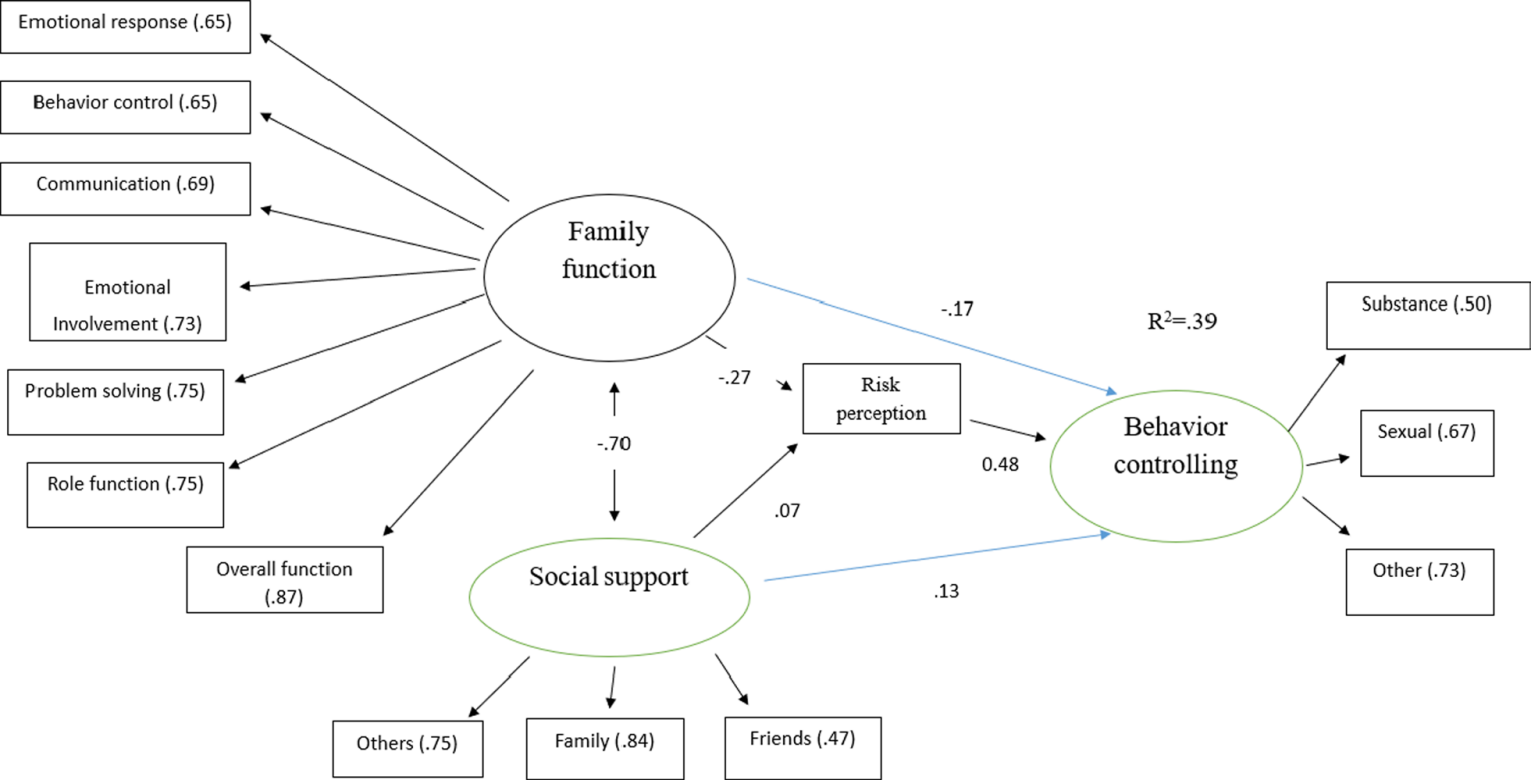
The Proposed Model of the Research for Investigating the Mediating Role of AIDS Risk Perception

The results of analyzing the model displayed in Fig. 1 showed that the three variables together, i.e., family functioning, perceived social support and AIDS risk perception, could predict 39% of the variance of controlling risky behavior associated with AIDS. Furthermore, the results indicated that family functioning with a standardized coefficient of − 0.17 (*p* < 0.009), perceived social support with a standardized coefficient of 0.13 (*p* < 0.04) and AIDS risk perception with a standardized coefficient of 0.47 (*p* < 0.01) could predict the variable of controlling risky behavior associated with AIDS.

The results demonstrated that the variables of family functioning, perceived social support and AIDS risk perception had direct effects on controlling risky behavior associated with AIDS, whereas only family functioning (β = − 0.13) had indirect effects on controlling risky behavior associated with AIDS (see Model 1 & Table 4).

**Table 4:** The results of path analysis for investigating the mediating role of AIDS risk perception

	Direct			Indirect		
	B	β	*p*	B	β	*p*
Family function → Risk perception	− 0.90	− 0.27	0.001			
Social support → Risk perception	0.25	0.07	0.25			
Risk perception → Behavior controlling	0.07	0.47	0.001			
Family function → Behavior controlling	− 0.08	− 0.17	0.009	− 0.06	− 0.13	0.001
Social support → Behavior controlling	0.07	0.13	0.04	0.022	0.03	0.282

B, regression weights; β, standardized regression weights

Additionally, the results showed that perceived social support had indirect effects on the subscales of controlling risky behavior associated with AIDS, so that the standardized coefficients of the subscales of drug use, risky sexual behavior and other behavior were 0.08, 0.11, and 0.12, respectively. It can be also stated that family functioning had indirect effects on the subscales of controlling risky behavior associated with AIDS, so that the standardized coefficients of the subscales of drug use, risky sexual behavior and other behavior were—0.15,—0.20, and—0.22, respectively.

### Model 2: investigate the mediating role of self-efficacy

**Fig. 2:**
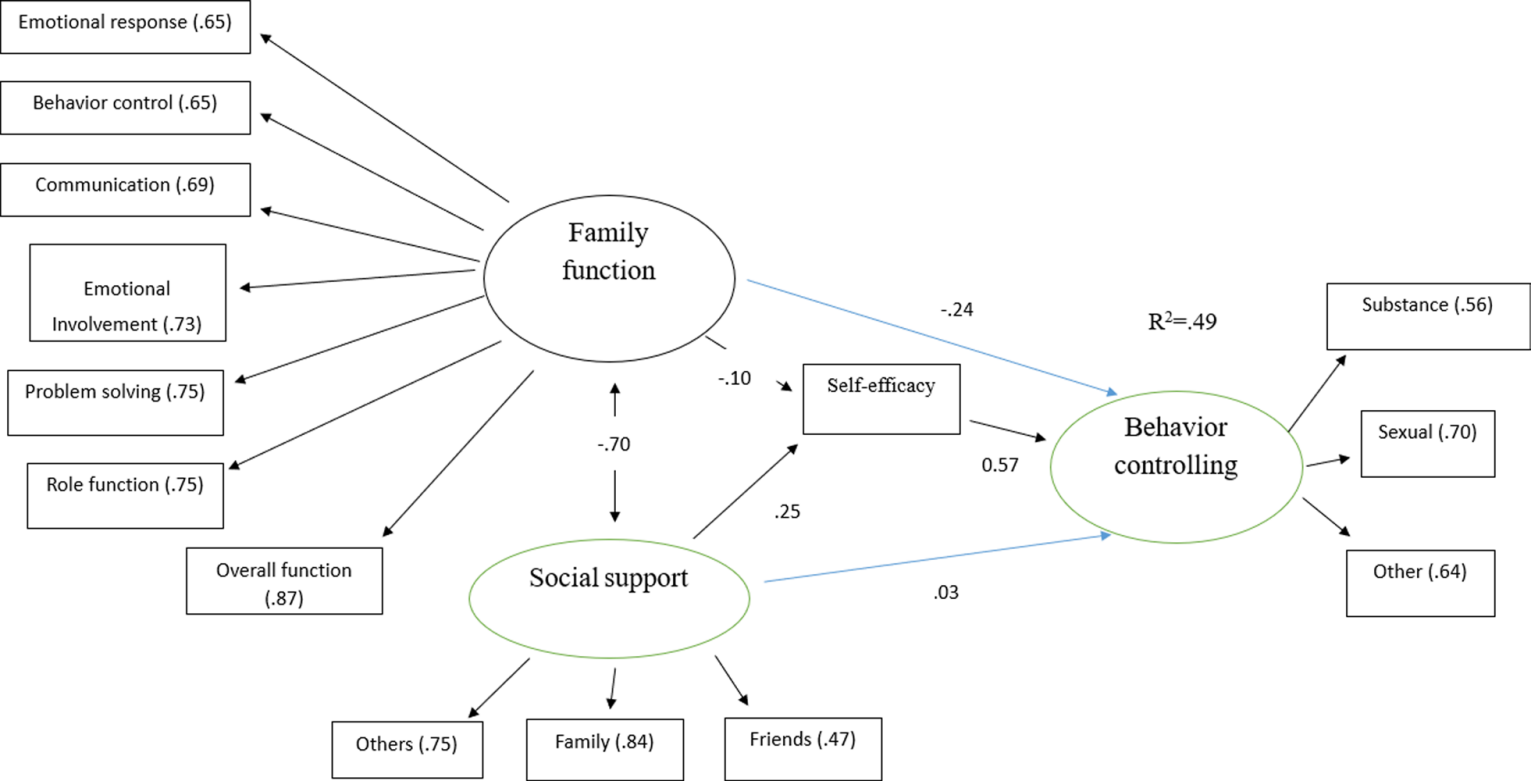
The Proposed Model of the Research for Investigating the Mediating Role of Self-efficacy in Controlling Risky Behavior

To investigate the mediating role of self-efficacy in controlling risky behavior associated with AIDS in the relationship of family functioning and perceived social support to controlling risky behavior associated with AIDS, a model was formulated (see Fig. 2).

The results of analyzing the model displayed in Fig. 2 demonstrated that the three variables together, i.e., family functioning, perceived social support and self-efficacy in controlling risky behavior associated with AIDS, could predict 49% of the variance of controlling risky behavior associated with AIDS. Additionally, the results showed that family functioning with a standardized coefficient of − 0.24 (*p* < 0.001) and self-efficacy in controlling risky behavior associated with AIDS with a standardized coefficient of 0.58 (*p* < 0.01) could predict the variable of controlling risky behavior associated with AIDS.

The results indicated that the variables of family functioning and self-efficacy in controlling risky behavior associated with AIDS had direct effects on controlling risky behavior associated with AIDS, whereas perceived social support did not have direct effects on controlling risky behavior associated with AIDS. Moreover, the results showed that family functioning did not have indirect effects on controlling risky behavior associated with AIDS (see Model 2 & Table 5), whereas perceived social support (β = 0.14) had indirect effects on controlling risky behavior associated with AIDS.

**Table 5:** The results of path analysis for investigating the mediating role of self-efficacy in controlling risky behavior

	Direct			Indirect		
	B	β	*p*	B	β	*p*
Family function → Self-efficacy	− 0.56	− 0.10	0.08			
Social support → Self-efficacy	1.55	0.25	0.001			
Self-efficacy → Behavior controlling	0.05	0.58	0.001			
Family function → Behavior controlling	− 0.12	− 0.24	0.001	− 0.02	− 0.06	0.091
Social support → Behavior controlling	0.02	0.03	0.591	0.08	0.14	0.001

On the other hand, the results indicated that perceived social support had indirect effects on the subscales of controlling risky behavior associated with AIDS, so that the standardized coefficients of the subscales of drug use, risky sexual behavior and other behavior were 0.10, 0.13, and 0.12, respectively. It can be also stated that family functioning had indirect effects on the subscales of controlling risky behavior associated with AIDS, so that the standardized indirect coefficients of the subscales of drug use, risky sexual behavior and other behavior were—0.17,—0.21, and—0.19, respectively.

The results of examining the fitness of the two models are shown in Table 6.

**Table 6:** The results of examining the fitness of the two models

Model	X^2^	df	GFI	IFI	CFI	RMSEA
1	279.40	72	0.95	0.95	0.95	0.062
2	290.99	72	0.95	0.95	0.95	0.063

*RMSEA* Root Mean Square Error of Approximation; *GFI* Goodness of fit index; *CFI* Comparative Fit Index; *IFI* Incremental Fit Index; *χ*^2^ Normed Chi-square; *df* Degrees of Freedom

The results shown in Table 6 are indicative of the fact that the two models have reasonable fitness because the values of RMSEA in all two models were lower than 0.08 [44].

## Discussion

The results of the present study demonstrated that family functioning, AIDS risk perception, and self-efficacy in controlling risky behavior associated with AIDS and controlling risky behavior associated with AIDS were related. Given that higher scores in family assessment devices (FAD) denote unhealthy functioning, i.e., the healthier the family functioning, the higher the perception of the members of the family of AIDS, they will have higher self-efficacy in controlling risky behavior and controlling risky behavior associated with AIDS are better conducted. Furthermore, the results of the present study showed that family functioning could predict the risky behavior associated with AIDS and AIDS risk perception, whereas it could not predict self-efficacy in controlling risky behavior associated with AIDS. Previous studies conducted in this field have been indicative of the role of family in displaying risky behavior associated with AIDS, including drug use and risky sexual behavior [15–21]. So, the results of this section of the study were consistent with those of previous studies.

The results of a systematic study demonstrated that the role of family structure has been confirmed in risky behavior in studies conducted in African countries over 2000–2014 [45].

However, some comparative studies showed that there were differences between the criminals’ families and peer groups [46, 47]. Some studies confirmed that there was a negative relationship between family functioning and criminal acts leading to prison [48–50]. Comparing the present work with previous studies in terms of the statistical population and analysis method indicates that family is a contributing factor in risky behavior, but the tools used for measuring family functioning and controlling risky behavior were different in these studies. In fact, what happens in a family and how it functions can be considered key factors in creating flexibility and reducing current and future risks associated with healthy living [51].

In another section, the results of the present study demonstrated that perceived social support, AIDS risk perception, self-efficacy in controlling risky behavior associated with AIDS and controlling risky behavior associated with AIDS were positively related. In other words, the higher one’s perceived social support, the extent of each of the other three factors will be greater.

As a further matter, the results indicated that perceived social support and family functioning together could predict controlling risky behavior associated with AIDS, but its impact factor was less than that of family functioning. The results of this section of the study were consistent with the preceding studies [4, 8, 9]. According to the findings of the present study, the higher self-efficacy is associated with the more support receives from family, friends and others. From the researchers’ points of view, the primary benefit of social support is the role that it plays in increasing one’s self-efficacy [52]. Chu (2010) believes that a family can support its members in two ways. First, it supplies its members with the necessary information and facilities, and second, it shares the existing emotions [53]. The same goes for risky behavior, too. If a family or friends can provide others with the needed information on AIDS and risky behavior, their risk perception will increase.

Moreover, the results of the present study showed that AIDS risk perception and controlling risky behavior associated with AIDS were positively related, i.e., higher risk perception will result in higher control over risky behavior. In the proposed model for predicting risky behavior based on family functioning and perceived social support, AIDS risk perception played a mediating role. In other words, it can be said that proper family functioning and high social support will lead to controlling risky behavior when one’s AIDS risk perception is high. The results of various studies have been indicative of the fact that low AIDS risk perception will give rise to risky sexual behavior [22, 23, 54], which was consistent with the results of the present study. However, it is noteworthy that the tool used in the present study to measure AIDS risk perception was different from those in previous studies. On the other hand, risk perception has been studied as an antecedent of behavior in some studies and it has been concluded that high risk perception would decrease risky behavior [55]. However, in some other studies, it has been studied as a mediator between personality and risky behavior [56], but the risk perception has not been dealt with in any earlier studies as a mediator between family functioning and risky behavior.

As a further matter, in the proposed model for predicting risky behavior based on family functioning and perceived social support, it was concluded that self-efficacy played a mediating role. The relationship between self-efficacy in controlling risky behavior associated with AIDS and AIDS preventive behavior has been confirmed in preceding studies, and this construct can play a major role in decreasing risky behavior leading to AIDS [29–31]. The results of a Taiwanese study showed that self-efficacy was the most powerful predictor of safe or unsafe sexual behavior [35]. Similarly, the results of an Iranian study revealed that, of the constructs of the health belief model, perceived self-efficacy could predict preventive and educational behavior associated with AIDS the most [34]. The results of a study revealed that ones with low self-efficacy in the face of abstaining from sexual contacts were exposed to sexual intercourse two times as much as those with high self-efficacy, and the level of condom use was lower fivefold [57]. In preceding studies, the role of self-efficacy has been confirmed as an important construct to reduce risky behavior associated with AIDS [30, 32, 33, 58]. The current study emphasizes the importance of the role of family in controlling risky behavior, and parents should promote self-efficacy in their children. We suggest that psychologists be educated about the risks of AIDS.

Furthermore, gender is one of the variables that plays a key role in the risk of AIDS. Gender roles in any society may organize relationships that play a key role in AIDS [59]. In the field of AIDS prevention, there is an increasing interest in the role of gender in the risk of AIDS, because educational programs can encourage men to engage in preventive behaviors [60]. AIDS-prevention programs will be most effective when they are gender-based, as social norms are different in male and female roles, and reduction in incidence rate of the disease is related to this consideration [61]. On the other hand, women play a vital role in society, but issues such as the economy and social system make them vulnerable to high-risk sex, which demonstrate the need to pay attention to women in the AIDS prevention program [62]. However, previous studies have shown that psychological interventions can be effective in promoting important variables in women's AIDS prevention [63]. Some researchers believe that safe sexual behavior in women is influenced by social and psychological factors such as self-efficacy [64, 65].

Iranian and Islamic cultures through religious beliefs, and teachings may influence the pattern of social relations especially in the AIDS/HIV disease. In Iran and other Islamic countries, there may be a belief that HIV is caused by extramarital relationships. Therefore, self-restraint from extramarital and amoral sexual behaviors is the important way to decrease the spread of AIDS/HIV. Moreover, Dadipoor (2020) claimed that AIDS is condemned among Iranian people [66]. ADIS is left unspoken because of religious beliefs. However, education as another important socio-cultural factor shaped the attitude towards AIDS. Thus, there is evidence that people with a high awareness about AIDS have a positive attitude towards this disease among Iranian population. Further, family function and social support as cultural factors can be an important barrier against HIV prevention [67]. For instance, one study found that perceived cultural norms lead to higher behavior controlling [68]. In general, Khodayari-Zarnaq et al. (2019) explained the cultural towards to ADIS in greater detail in which four components occur sequentially. (1) Traditional culture such as social obscenity, untouched, and unspoken; (2) religion variable such as taboo and big sin; (3) specific cultural and societal circumstances for example sexual issues, social support and educational consideration; and (4) addiction to drugs [69].

However, in Iran, the role of cultural factors in prevention of AIDS are still in an early stage. It follows that sociocultural factors are also a new issue and needs more investigation. Given the lack of reliable information on the status of people with ADIS, there is a great need for research and most importantly for interventions to prevention and to cope with knowledge related to this disease. Therefore, those interventions have to be culturally sensitive.

## Conclusion

According to the results, it can be said that self-efficacy is a mediating variable in the relationship of family functioning and perceived social support to controlling risky behavior. Therefore, it is recommended that families and psychologists promote self-efficacy in order to prevent the occurrence of high-risk behaviors.

Given that the present study was conducted in Iran, extreme caution must be exercised when generalizing the results and these models must be investigated in other societies, too. Hence, it is better to take this point into consideration when generalizing the results. Since our study was conducted on Iranian young’s, future research should be conducted on other samples. This study was unique and the results was interesting, so we can use the results across prevention of AIDS.

## Data Availability

The datasets used during the current study are available from the corresponding author on reasonable request.
